# Long-term infusion of nesfatin-1 causes a sustained regulation of whole-body energy homeostasis of male Fischer 344 rats

**DOI:** 10.3389/fcell.2015.00022

**Published:** 2015-04-08

**Authors:** Sima Mortazavi, Ronald Gonzalez, Rolando Ceddia, Suraj Unniappan

**Affiliations:** ^1^Department of Veterinary Biomedical Sciences, Western College of Veterinary Medicine, University of SaskatchewanSaskatoon, SK, Canada; ^2^School of Kinesiology and Health Sciences, York UniversityToronto, ON, Canada

**Keywords:** nesfatin-1, food intake, body weight, physical activity, energy expenditure, osmotic mini-pumps, metabolism, rats

## Abstract

Nesfatin-1, the N-terminal fragment of nucleobindin 2 (NUCB2), is an 82 amino-acid peptide that inhibits food intake and exerts weight-reducing effects. Nesfatin-1 has been proposed as a potential anti-obesity peptide. However, studies to date have mainly focused on the acute satiety effects of centrally administered nesfatin-1. The main objective of our studies was to characterize the long-term/chronic effects of peripheral administration of nesfatin-1 on whole-body energy balance and metabolic partitioning in male Fischer 344 rats. Short-term (1 day) subcutaneous infusion of nesfatin-1 (50 μg/kg body weight/day) using osmotic mini-pumps increased spontaneous physical activity and whole-body fat oxidation during the dark phase. This was accompanied by decreased food intake and basal metabolic rate compared to saline infused controls. On the seventh day of nesfatin-1 infusion, cumulative food intake, and total spontaneous physical activity during the dark phase were significantly reduced and elevated, respectively. Meanwhile, intraperitoneal injection of nesfatin-1 only caused a dark phase specific reduction in food intake and an increase in physical activity. NUCB2 mRNA expression in the brain and stomach, as well as serum NUCB2 concentrations were significantly reduced after 24 h fasting, while a post-prandial increase in serum NUCB2 was found in *ad libitum* fed rats. Collectively, our results indicate that chronic peripheral administration of nesfatin-1 at the dose tested, results in a sustained reduction in food intake and modulation of whole body energy homeostasis.

## Introduction

Nesfatin-1 (NEFA/nucleobindin 2-Encoded Satiety-and FAT-Influencing protein-1) is an 82 amino acid cleavage product of the precursor protein nucleobindin 2 (NUCB2) (Oh-I et al., 2006). NUCB2 is expressed in the hypothalamic paraventricular nucleus (PVN), arcuate nucleus (ARC), lateral hypothalamus (LH), supraoptic nucleus (SON), and zona incerta (ZI) along with the nucleus of the solitary tract (NTS) in rodents. Nesfatin-1 was also found in rat cerebrospinal fluid (Oh-I et al., 2006). In addition to hypothalamus nuclei and NTS, NUCB2 positive cells were localized in the brain stem regions consisting of Edinger Westphal (EW), dorsal motor of nucleus of vagus (DMNV), and caudal raphe nuclei of rats (Oh-I et al., 2006; Brailoiu et al., 2007; Foo et al., 2008). Double-labeling immunohistochemistry revealed that NUCB2 staining cells co-localize with vasopressin, oxytocin, cocaine and amphetamine regulated transcript (CART), and melanin concentrating hormone (MCH) (Kohno et al., 2008). Cholecystokinin (CCK) activates NUCB2 neurons in the PVN and NTS through a corticotropin-releasing factor (CRF)2-receptor-dependent pathway (Stengel et al., 2009). The presence of NUCB2 in brain regions known to regulate energy balance and body weight (Oh-I et al., 2006; Brailoiu et al., 2007; Foo et al., 2008) suggest a possible role for NUCB2 derived peptides in the regulation of energy balance.

In agreement with this notion, several studies have indicated that nesfatin-1 has anorectic effects in rats and mice. In fact, intracerebroventricular injection of nesfatin-1 reduces dark phase feeding and overall body weight in a dose dependent manner (Oh-I et al., 2006; Stengel et al., 2009; Gonzalez et al., 2011; Wernecke et al., 2014). The satiety induced by nesfatin-1 was found independent of leptin, however, central administration of alpha-MSH has been shown to increase NUCB2 mRNA expression in the hypothalamus (Oh-I et al., 2006). It was also shown that only nesfatin-1, not nesfatin-2 or 3, the other two fragments of NUCB2 exerts anorectic effects in rodents (Oh-I et al., 2006). Similar to the effects seen in response to central administration, intraperitoneal injections of nesfatin-1 also caused a remarkable suppression of food intake in lean and obese mice (Shimizu et al., 2009). However, this effect lasted for only 1 h after injection. It was followed by an increase in food intake in nesfatin-1 treated animals. Peripheral administration of nesfatin-1 reduced nocturnal food intake in rats (Stengel et al., 2009; Gonzalez et al., 2011; Wernecke et al., 2014). The satiety effects of nesfatin-1 are blocked by the co-injection of CRF-receptor antagonists, suggesting that nesfatin-1 may act through hypothalamic CRF (Stengel et al., 2009). Nesfatin-1 crosses the blood-brain barrier (BBB) without saturation, which could be of clinical significance for its use as a pharmacological agent for obesity treatment (Pan et al., 2007; Price et al., 2007). These findings suggest central effects of nesfatin-1 in regulating metabolism.

There are several reports available now indicating the metabolic effects of nesfatin-1 in rats and mice (Gonzalez et al., 2012; Mohan and Unniappan, 2013). However, most of these experiments were restricted to short-term, central effects of nesfatin-1 on food intake (Oh-I et al., 2006; Shimizu et al., 2009; Stengel et al., 2009, 2012; Atsuchi et al., 2010; Yosten and Samson, 2010; Goebel et al., 2011; Chen et al., 2012; Konczol et al., 2012; Gotoh et al., 2013; Wernecke et al., 2014). Some exceptions are Shimizu et al. (2009) and Dong et al. (2014), who employed peripheral injections to administer nesfatin-1. Li et al. (2013) examined the effects of continuous peripheral infusion of nesfatin-1 on glucose homeostasis, food intake, and body weight. We provided the first comprehensive metabolic profiling of peripherally administered nesfatin-1 effects in rats (Gonzalez et al., 2011). However, this study also just monitored the effects of nesfatin-1 for 5 h post-infusion during the dark phase. Given the short half-life of nesfatin-1 in circulation (Pan et al., 2007; Price et al., 2007), acute peripheral injections may not sufficiently induce nesfatin-1 related metabolic functions in rodents. In addition, from a therapeutic perspective, continuous peripheral administration of nesfatin-1 would be the preferred mode of delivery for the treatment for metabolic diseases, especially obesity. To the best of our knowledge, there are no reports on the chronic effects of peripherally administered nesfatin-1 on energy balance. This study extends our own previous findings, and tests a previously unknown aspect of nesfatin-1 effects on energy balance. Using automated metabolic cages, we carried out an in-depth and comprehensive analysis of the metabolic effects of continuous peripheral administration of nesfatin-1 in lean rats. While our results confirm the previously known anorectic effects of nesfatin-1, it also provides novel information on sustained effects of nesfatin-1 on whole body energy homeostasis. Circulating levels of nesfatin-1/NUCB2 increased post-meal, indicating a meal responsive secretion of this metabolic hormone. In addition, we found that fasting reduces NUCB2 mRNA expression in the brain and stomach and reduces circulating levels of nesfatin-1, while re-feeding reverses these changes.

## Materials and methods

### Animals

Unless otherwise specified, age and weight (~200 g) matched lean, male Fischer 344 rats purchased from Charles River Laboratories Inc. (Saint-Constant, Quebec, Canada) were used for all studies. Research protocols used in this study adhered to the guidelines of the Canadian Council for Animal Care and was approved by the York University Animal Care Committee (Protocol number 2007-5). Rats were individually housed in polycarbonate cages with bedding in a 12 h light (0700–1900 h):12 h dark (1900–0700 h) photoperiod at 23 ± 1°C and controlled humidity at the York University vivarium. Animals had *ad libitum* access to tap water and normal rat chow (Purina Mills, St. Louis, Missouri).

### Materials

The 82 amino acid rat nesfatin-1 (VPIDVDKTKVHNVEPVESARIEPPDTGLYYDEYLKQVIEVLETDPHFREKLQKADIEEIRSGRLSQELDLVSHKVRTRLDEL) was synthesized by Abgent Technologies (San Diego, California) and was purchased through MJS Biolynx (Toronto, Ontario, Canada). Synthetic nesfatin-1 was HPLC purified to 95% purity, and the mass and purity were confirmed by mass spectrometry. Nesfatin-1 was freshly prepared by dissolving in 0.9% sterile saline (0.9% sodium chloride) for each study and the resuspended peptide never underwent freeze-thaw cycles. Sterile saline was purchased from Medical Mart Inc. (Toronto, Ontario, Canada). One-day (Model 2001D) and 7-day (Model 2ML1) Alzet™ osmotic mini-pumps were purchased from Durect Corporation (Cupertino, California).

### Animal acclimation, surgery, and monitoring

It has been shown that acclimation is critical in reducing handling stress, and in obtaining consistent results on food intake (Abbott et al., 2006). The acclimation protocol used here was validated for our research, and was reported earlier (Unniappan et al., 2006; Unniappan and Kieffer, 2008; Gonzalez et al., 2011). Briefly, rats were housed in polycarbonate cages for 7 days from the day of arrival and then routinely handled for 7 days before starting the experiment. From the first day of acclimation, animals were transported to the surgery room, gently handled for several minutes and returned to their respective cages. On day 3 of acclimation, rats were anesthetized using 3% isoflurane and oxygen as the gaseous carrier and shaved in the area where the implantation was to be made. On the implantation day, weight matched rats (~200 g) were anesthetized, a small sub-clavicular incision made and osmotic mini-pumps were implanted subcutaneously. Following implantation, wounds were immediately sealed using wound clips (VWR, Canada) and antiseptics were applied using cotton swabs. Rats were then removed from the anesthetic machine, weighed and returned to their cages and allowed to recover from anesthesia in their cages. For automated monitoring of feeding and other parameters, animals were transferred to the Comprehensive Laboratory Animal Monitoring System (CLAMS; Columbus Instruments, Ohio) cages. Prior to monitoring *in vivo* metabolic variables, the CLAMS gas sensors and balances were calibrated following manufacturer's guidelines. After implantation of the pumps (always completed an hour before the dark phase), the animals were placed in CLAMS cages equipped to provide continuous automated monitoring of water and food intake as well as locomotor activity. Each cage was also connected to an open-circuit calorimeter for determination of oxygen consumption (VO_2_), CO_2_ production (VCO_2_), respiratory quotient (RQ), and energy expenditure (EE). Animals were allowed to acclimate to the CLAMS cages for an hour post-surgery and following this, all variables were recorded for 24 h (1800–1900 h next day).

The equations used for all calculations are provided in the Columbus Instruments website (http://www.colinst.com/brief.php?id=51). RQ was calculated by dividing VCO_2_ by VO_2_ with values ranging from 0.7 to 1.0. A shift of RQ values toward 0.7 indicates that fatty acids contribute the most to energy production, while a shift toward 1.0 indicates that carbohydrates are the main substrates used. EE was determined using the following equation: EE = CV ^*^ VO_2_, where the calorific value is multiplied by VO_2_. CV was determined using the following equation: CV = (3.815 + 1.232) ^*^ RQ. Each cage is also equipped with a system of infrared beams that detects animal movement in the X (the length of the cage) and Z (the height of the cage) axes. The energy derived from carbohydrates and fats were extrapolated from the heat/liter data obtained from CLAMS, using the methods of McLean and Tobin, as described in the protocols provided by Columbus Instruments. The activities were presented as total horizontal (X-TOT), ambulatory (X-AMB, which refers to successive beam breaks in the X axis), and total vertical (Z-TOT) movements. Acclimated rats were used in all studies and same rats were never used for more than one study.

### Assessment of metabolic effects of short-term (1 day) continuous infusion of nesfatin-1

Fischer 344 rats (*n* = 4/group; study repeated twice) were acclimated and implanted with osmotic mini-pumps containing synthetic rat nesfatin-1 (50 μg/kg body weight/day) or saline and were monitored using CLAMS as described above. We conducted pilot studies where nesfatin-1 at doses 1, 10, 50, and 100 μg/kg body weight/day were infused using osmotic mini-pumps to male Fischer 344 rats. From these studies, we selected a dose (50 μg/kg body weight/day or ~5 nmol/kg BW/day) of nesfatin-1 that was effective in reducing food intake (Gonzalez et al., 2011). The dose of nesfatin-1 tested here is lower compared to the 0.5 and 1.25 nmol/g doses of nesfatin-1 used earlier in an intraperitoneal injection study (Shimizu et al., 2009). The experiment was repeated twice using different sets of rats, with same results obtained. A representative set of data from one experiment (*n* = 4 rats/group) is provided here.

### Evaluation of metabolic effects of long-term (7 day) continuous infusion of nesfatin-1

To test the long-term effects of nesfatin-1 on rats, in a separate study, a 7-day continuous infusion of nesfatin-1 was employed. Rats (*n* = 4/group; study repeated twice) were implanted with 7-day osmotic mini-pumps infusing 50 μg/kg body weight/day of nesfatin-1. Daily cumulative food intake and body weight were manually determined daily. Using the CLAMS, complete metabolic activities of the saline (control, *n* = 4) and nesfatin-1-infused (*n* = 4) rats were measured over 24 h (1800–1800 h next day) on days 6–7 during the course of a 7-day infusion-period. This experiment was also repeated twice, and representative data (*n* = 4 rats/group) from one experiment are provided.

### Effects of acute administration of nesfatin-1 on whole body energy homeostasis

Rats (*n* = 4/group) were intraperitoneally (i.p.) injected with, 200 μL of 0.9% sterile saline (sodium chloride, control) or 50 μg/kg body weight/day of nesfatin-1 dissolved in sterile saline, using a 27-gauge needle attached to a 1 mL syringe (Becton-Dickinson, Ontario, Canada). CLAMS measurements were conducted as described earlier.

### Investigations on the effects of feeding status of rats on NUCB2 mRNA expression in the brain and stomach

Since nesfatin-1 is an anorexigen (Oh-I et al., 2006), it is possible that the NUCB2 mRNA display a meal-regulated pattern in its expression in the brain and stomach, two major tissue sources of NUCB2/nesfatin-1. NUCB2 immunoreactivity has been reported in a large number of brain regions (Oh-I et al., 2006; Brailoiu et al., 2007; Foo et al., 2008; Kohno et al., 2008; Stengel et al., 2008; Goebel et al., 2009), therefore, total RNA was extracted from whole brain. To determine whether NUCB2 mRNA expression is altered during fasting, brain and stomach tissues were collected from *ad libitum* fed rats, and rats deprived of food for 24 h. Briefly, animals were euthanized by inhalation of an overdose of isoflurane, followed by cervical dislocation. Brain (whole tissue), and stomach (whole stomach) were removed, immediately frozen in liquid nitrogen, and then stored at −80°C until total RNA was extracted using TRIzol™ Reagent (Invitrogen Canada Inc., Ontario, Canada) or Aurum total RNA kit (Bio-Rad laboratories Inc., Ontario, Canada) according to the manufacturer's instructions. Optical density readings at 260 nm were made to determine the total RNA concentration of each tissue sample using spectrophotometer (MultiSKAN SPECTRON, Thermo Fisher Scientific Inc., Canada). RNA purity was determined through the 260/280 OD ratio. Total RNA was stored at −80°C. cDNA was synthesized using iScript cDNA synthesis kit (Bio-Rad laboratories, Inc. Ontario, Canada) based on the manufacturer's instructions and were kept at −20°C until used for quantitative real-time (qRT) PCRs. For quantification, rat NUCB2 forward primer (5′-GCTG TTCCTATTGATGTGG-3′) and reverse primer (5′-CTTCCTACTTCTTGCCTC-3′) were used.

### Effects of the feeding status on serum nesfatin-1/NUCB2 levels

If nesfatin-1 is indeed an endogenous anorexigen, we should see an increase in circulating levels of nesfatin-1 following a meal and a decrease under fasting conditions. In order to determine any meal related changes in circulating nesfatin-1/NUCB2, we collected serum samples from *ad libitum* fed rats and rats deprived of food for 24 h. In addition, serum was also collected from rats an hour prior to the commencement of their feeding (1800 h) and 3 h after starting their active feeding (2200 h). Nesfatin-1/NUCB2 ELISA was conducted as described previously by us (Gonzalez et al., 2011).

### Statistical analyzes

Statistical analyses for quantitative real-time PCR, CLAMS studies, and tissue mass comparison were conducted using Two-Way ANOVA followed by Newman-Keuls post-test (GraphPad Prism 4, San Diego, USA). Mann-Whitney test or Student's *t*-tests were used for testing groups within the CLAMS, mRNA expression and ELISA data. Significance was considered at *P* < 0.05. Data are expressed as mean + SEM.

## Results

### Short-term (1 day) nesfatin-1 treatment caused dark-phase specific metabolic changes

**Figure 1:**
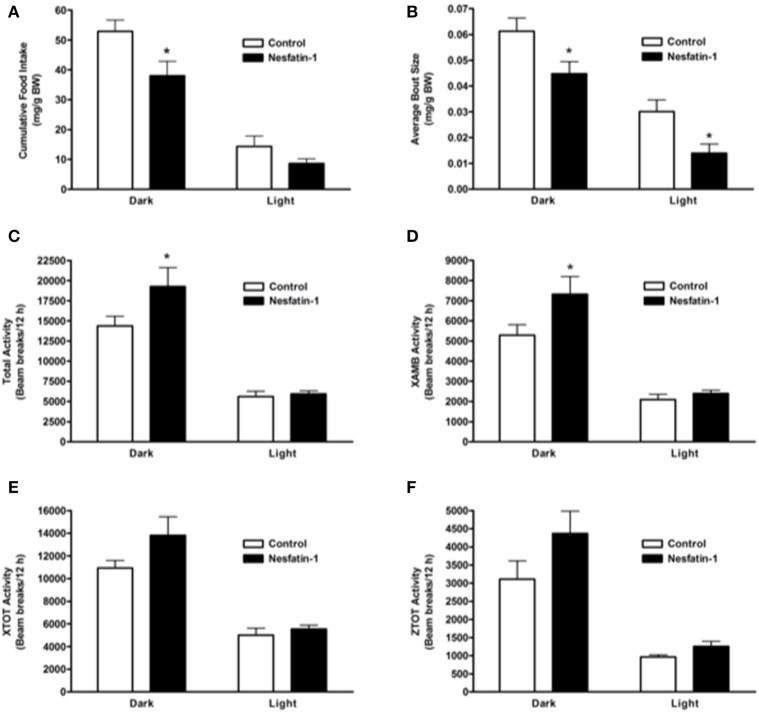
**Cumulative food intake (mg/g BW; A) and average feeding bout size (mg/g BW; B) decreased in rats continuously infused with 50 μg/kg body weight/day nesfatin-1 for 1 day**. An increase in locomotor activity (beam breaks/12 h; **C–F**) was also observed during the dark phase of nesfatin-1 infusion. The activities were presented as total horizontal (X-TOT), ambulatory (X-AMB, which refers to successive beam breaks in the X axis), and vertical (Z-TOT) movements. Dark cycle occurred during 1900 to 0700 h, while the light cycle was from 0700 to 1900 h next day. All data are presented as means ± SEM with an *n* = 4 rats/group. ^*^P < 0.05 compared to control.

When compared to controls, nesfatin-1 treatment elicited a significant reduction in cumulative food intake during the 12-h dark phase (Figure 1A). The average feeding bout size was significantly lower for nesfatin-1 treated animals compared to saline treated controls in both dark and light phases (Figure 1B). Total (Figure 1C), ambulatory (Figure 1D), horizontal (Figure 1E) and vertical (Figure 1E) activities of nesfatin-1 treated rats were significantly higher than control group during the dark phase. The spontaneous physical activity was not different in nesfatin-1-treated and control animals during the light phase (Figures 1C–F). The total number of feeding bouts was not significantly different between treated and untreated animals (Supplemental Figure 1A). No change in cumulative water intake was observed between nesfatin-1 treated and control animals (Supplemental Figure 1B). There were no significant differences in the body weight of nesfatin-1 treated and control animals (Supplemental Figure 1C).

**Figure 2:**
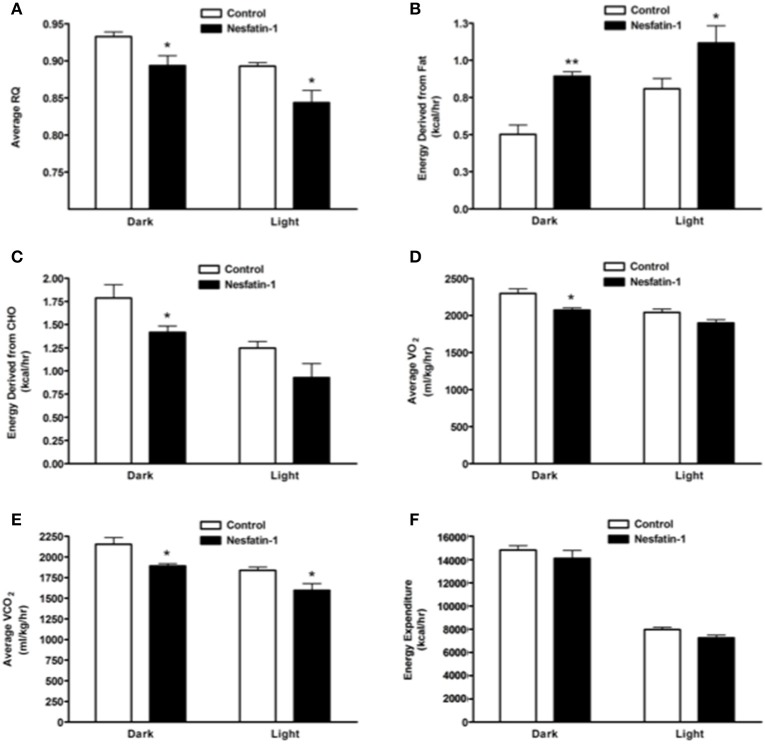
**Respiratory quotient (RQ; A) and relative contribution of fatty acids (B) and carbohydrates (C) to energy expenditure, average O_2_ consumption (D) and CO_2_ production (E) were altered during 1 day continuous infusion of nesfatin-1**. No changes in energy expenditure **(F)** were observed during the treatment. The dark cycle occurred from 1900 to 0700 h, while the light cycle was from 0700 to 1900 h next day. Data are represented as means ± SEM with an *n* = 4 rats/group. ^*^P < 0.05, ^**^P < 0.01 compared to control.

Average RQ of nesfatin-1 treated rats showed significant reduction in comparison with controls in both dark and light phases (Figure 2A). The relative contribution of fatty acids toward total energy production was significantly higher in nesfatin-1 treated rats during both dark and light phases (Figure 2B). On the other hand, carbohydrate oxidation was significantly reduced during the dark phase alone (Figure 2C). Oxygen consumption (VO_2_) was significantly lower in nesfatin-1 treated rats during the dark phase (Figure 2D), and CO_2_ production was also reduced in nesfatin-1 treated rats compared to the saline controls during the dark and light phases (Figure 2E). No change in total energy expenditure was observed between nesfatin-1 treated animals and saline controls (Figure 2F).

### Long-term nesfatin-1 treatment resulted in lowering of food intake and an increase in physical activity

**Figure 3:**
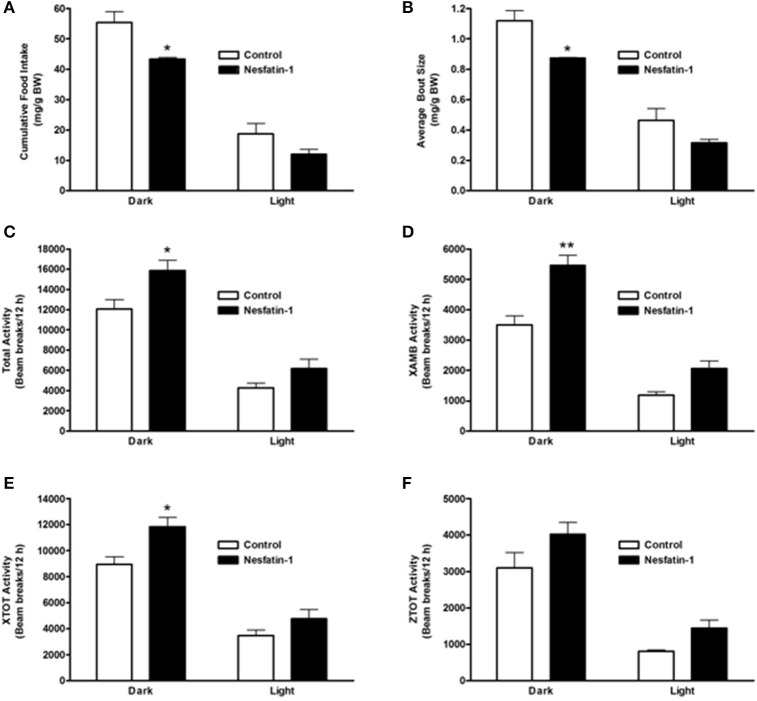
**Cumulative food intake (mg/g BW; A) and average feeding bout size (mg/g BW; B) of rats remained reduced on the seventh day of continuous infusion with nesfatin-1**. Increases in locomotor activity were also observed in nesfatin-1 treated animals (beam breaks/12 h; **C–F**) during the seventh day of continuous infusion. The activities were presented as total horizontal (X-TOT), ambulatory (X-AMB, which refers to successive beam breaks in the X axis), and vertical (Z-TOT) movements. Dark cycle occurred during 1900 to 0700 h, while the light cycle was from 0700 to 1900 h next day. All data are presented as means ± SEM with an *n* = 4 rats/group. ^*^P < 0.05 compared to control, and ^**^P < 0.01 compared to controls.

On the last day (day 7) of continuous infusion of nesfatin-1, cumulative food intake during the dark phase was significantly reduced compared to the controls (Figure 3A). Similar to the short-term study, the average size of feeding bouts was significantly smaller in nesfatin-1 treated animals compared to controls during the dark phase (Figure 3B). Total activity (XTOT + ZTOT) remained significantly higher (~32%) for nesfatin-1 treated animals compared to saline treated controls (Figure 3C). Similarly, total ambulatory activity (XAMB) on the seventh day of infusion remained higher (~56%) in nesfatin-1 treated rats than control rats (Figure 3D). Nesfatin-1 treated rats experienced higher (~32%) than control nocturnal total horizontal activity on the seventh day of continuous infusion (Figure 3E). The number of dark phase feeding bouts of nesfatin-1 treated rats was not different than saline control rats on the seventh day of infusion (Supplemental Figure 2A). No differences were found in the water intake (Supplemental Figure 2B), total vertical activity (Figure 3F), and body weight (Supplemental Figure 2C) of saline treated and nesfatin-1 treated rats after 7 days of chronic infusion.

**Figure 4:**
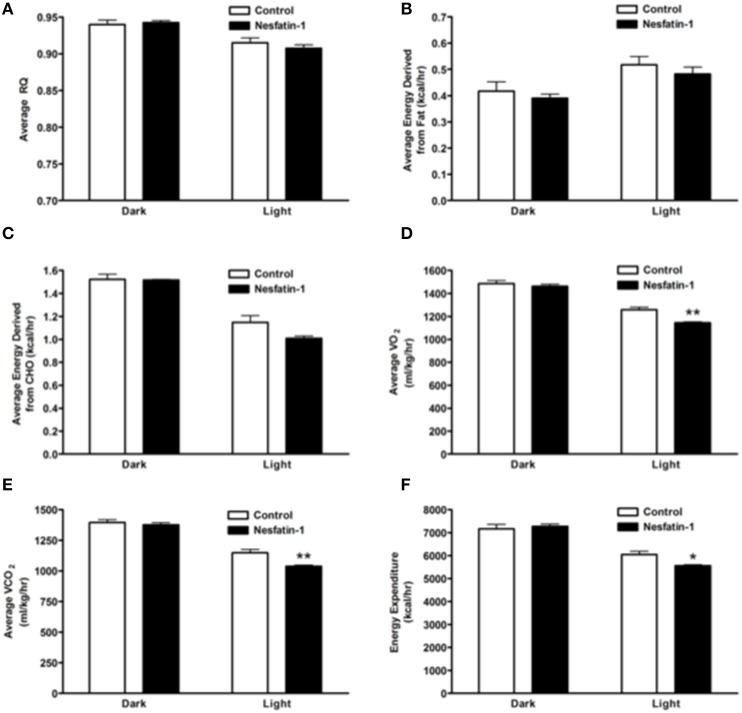
**Respiratory quotient (A) and relative contribution of fatty acids (B) and carbohydrates (C) to energy expenditure was not altered on day 7 of continuous infusion of nesfatin-1**. However, the effect of nesfatin-1 on average O_2_ consumption **(D)**, CO_2_ production **(E)**, and energy expenditure **(F)** was reduced during the light phase of the seventh treatment day. The dark cycle in each panel occurred from 1900 to 0700 h, while the light cycle was from 0700 to 1900 h next day. Data are represented as means ± SEM with an *n* = 4 rats/group. ^*^P < 0.05, ^**^P < 0.01 compared to control.

The RQ of nesfatin-1 treated rats were not different from control rats at the end of the 7-day-nesfatin-1-infusion period (Figure 4A). The relative contribution of either carbohydrates or fatty acids toward energy production in nesfatin-1 treated rats was not statistically different between groups on the seventh day of the infusion period (Figures 4B,C). Interestingly, both average O_2_ consumption and average CO_2_ production decreased during the light phase for nesfatin-1 treated rats during the seventh day of infusion (Figures 4D,E). We observed a significant reduction in energy expenditure during the light phase in nesfatin-1-treated animals (Figure 4F) compared to saline treated controls. However, the total energy expenditure of nesfatin-1 treated rats during the dark phase was same as in control rats (Figure 4F).

### Acute administration of nesfatin-1 decreases food intake and increases physical activity during the dark phase

**Figure 5:**
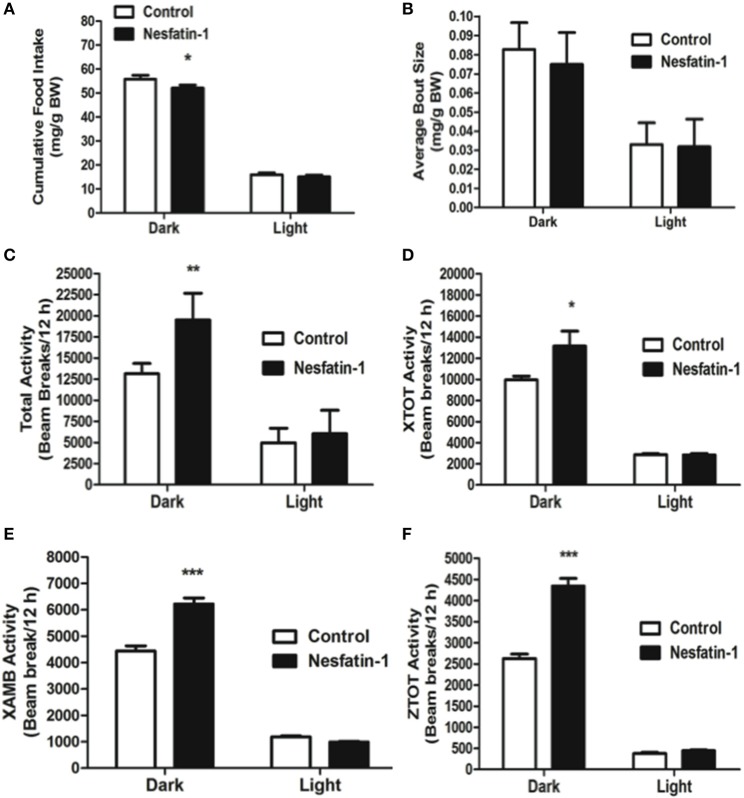
**Cumulative food intake (mg/g BW; A) was significantly reduced, but the average feeding bout size (mg/g BW; B) was not influenced by nesfatin-1 during the dark phase**. Increases in locomotor activity were also observed in nesfatin-1 treated animals (beam breaks/12 h; **C–F**) during the dark phase. The activities were presented as total horizontal (X-TOT), ambulatory (X-AMB, which refers to successive beam breaks in the X axis), and vertical (Z-TOT) movements. Dark cycle occurred during 1900 to 0700 h, while the light cycle was from 0700 to 1900 h next day. All data are presented as means ± SEM with an *n* = 4 rats/group. ^*^P < 0.05 compared to control, ^**^P < 0.01 compared to control, ^***^P < 0.001 compared to control.

**Figure 6:**
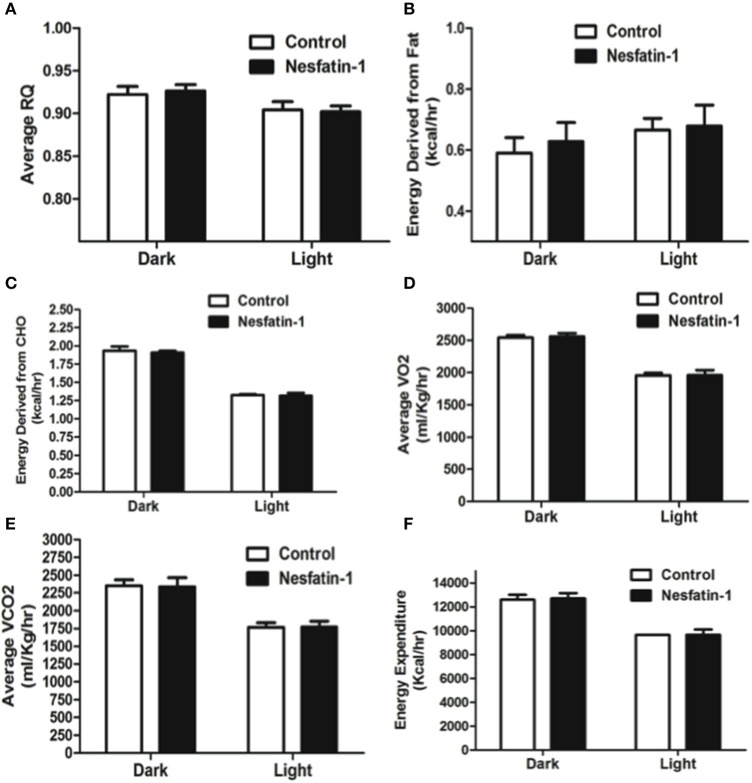
**Respiratory quotient (A) and relative contribution of fatty acids (B) and carbohydrates (C) to energy expenditure, average O_2_ consumption (D), CO_2_ production (E), and energy expenditure (F) were not altered as a result of nesfatin-1 infusion**. The dark cycle in each panel occurred from 1900 to 0700 h, while the light cycle was from 0700 to 1900 h next day. Data are represented as means ± SEM with an *n* = 4 rats/group.

Intraperitoneal injection of nesfatin-1 caused a dark phase specific reduction in food intake (Figure 5A), and physical activity (Figures 5C–F). Unlike the day 1 and day 7 results, nesfatin-1 was found to have no effects on other metabolic parameters tested (Figures 5B, 6A–F).

### NUCB2 mRNA expression was reduced during fasting and increased after re-feeding, while serum nesfatin-1/NUCB2 levels are elevated after a post-meal, and reduced after food deprivation

**Figure 7:**
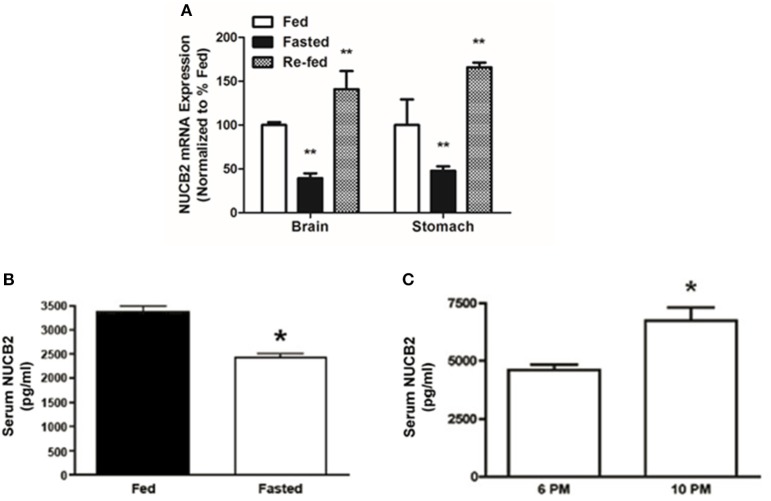
**Fasting for 18 h reduces NUCB2 mRNA expression in the brain and stomach (A) of rats**. Re-feeding for 4 h normalized NUCB2 mRNA expression in the brain **(A)**. Serum NUCB2 levels were lower in rats fasted for 18 h **(B)**. There was a post-prandial increase in serum nesfatin-1/NUCB2 levels in rats 3 h after the beginning of dark phase **(C)**. All data are presented as means ± SEM. *n* = samples from six rats analyzed in duplicates. ^*^P < 0.05 compared to fed rats or at 6 p.m., ^**^P < 0.01 compared to unfed rats.

Compared to the expression levels in *ad libitum* fed controls, NUCB2 mRNA expression in the brain and stomach was significantly reduced (~60%) after food deprivation for 24 h (Figure 7A). Re-feeding for 3 h after the 24-h food deprivation resulted in a return of brain NUCB2 mRNA expression to that in the brain of fed rats (Figure 7A). Serum nesfatin-1/NUCB2 levels in rats deprived of food for 24 h were significantly lower compared to that in *ad libitum* fed rats (Figure 7B). Levels of circulating nesfatin-1/NUCB2 significantly increased upon feeding (at 2200 h; 3 h after commencing the dark phase), compared to that found at a time immediately prior to the dark phase (at 1800 h; final hour of the light phase) (Figure 7C).

## Discussion

While some of the research reported here are confirmatory, it also provides insights on several previously uncharacterized aspects of nesfatin-1 on metabolism in rats. We report here that peripherally administered nesfatin-1 causes a sustained reduction in food intake in Fischer 344 rats. Continuous, peripheral (sub-cutaneous) infusion of nesfatin-1 significantly reduced food intake during the dark phase in our short-term (1 day). These results obtained using automated metabolic cages that avoided potential effects of human interference, confirms previous findings on the anorectic actions, especially dark phase specific food intake reducing actions of nesfatin-1 in rodents (Oh-I et al., 2006; Shimizu et al., 2009; Gonzalez et al., 2011; Konczol et al., 2012; Wernecke et al., 2014). Further, our results indicate that nesfatin-1 sustain its anorectic effect during a 7 day infusion period. While we did not see any differences in the duration of feeding bouts, nesfatin-1 treatment caused a reduction in the average bout size. The net reduction in food intake is likely achieved by the cumulative reductions in feeding bouts during the dark phase. It was reported that nesfatin-1 crosses the BBB in a bidirectional manner (Pan et al., 2007; Price et al., 2007). Therefore, it is possible that peripherally administered nesfatin-1 reaches the brain to elicit its effects. In addition, it was demonstrated that nesfatin-1 can activate vagal afferent neurons by stimulating calcium influx through N-type channels, thus conveying an indirect mechanism by which peripheral nesfatin-1 can relay a satiety signal to the brain (Iwasaki et al., 2009). Previous research has shown that nesfatin-1 can remain stable in the serum for a period of 10 min (Price et al., 2007), although Pan et al. (2007) have shown that nesfatin-1 has a relatively long half-life of ~20 min in both blood and brain. Furthermore, nesfatin-1 diffuses bi-directionally through the BBB using a non-saturable mechanism (Pan et al., 2007; Price et al., 2007). It is also likely that nesfatin-1 inhibits feeding and influences feeding behavior by affecting gut motility and transit time. However, further studies are required to explore these possibilities.

The reason for the transient dark phase satiety effects of nesfatin-1 is also not completely understood, although, previous studies have shown that nesfatin-1-induced satiety is not due to taste aversion (Shimizu et al., 2009). Stengel et al. (2009) provides pharmacological evidence that the nesfatin-1 induced dark-phase inhibition of feeding is mediated by the activation of CRF2 receptors in the hypothalamus. The mechanisms of action of nesfatin-1 and the pathways that mediate nesfatin-1 induced satiety are topics that require further investigations. To date, one study has reported the effects of acute peripheral administration of nesfatin-1 on food intake which carefully titrated the IC50 of this effect to 300 nmol/kg bw in rats (Shimizu et al., 2009). However, at present the receptor(s) for nesfatin-1 both centrally and peripherally remain unknown and any conclusions of an IC50 based on a single parameter (food intake) can only be construed as speculation.

We also found that nesfatin-1 reduced average O_2_ consumption and CO_2_ production during both dark and light phases. This resulted in a decrease in RQ, which indicated a shift in substrate partitioning. In fact, lipid oxidation was significantly increased in nesfatin-1-treated animals during both dark and light phases in the 1 day-study. Interestingly, total EE was not different between control and nesfatin-1-infused rats, despite increased spontaneous physical activity during the dark phase in rats treated with nesfatin-1. This indicates that energy-sparing mechanisms must have been activated to compensate for the increased physical activity observed in nesfatin-1 treated rats. It is also important to consider that nesfatin-1 effects observed in our study were dependent on the species studied, mode of delivery, and the doses tested.

A potential explanation for the increased dark-phase physical activity could reside in the fact that nesfatin-1 treatment elicits behaviors including anxiety and fear (Merali et al., 2008). In fact, Yosten and Samson (2009) have reported that acute central administration of nesfatin-1 causes hyperactivity that recovers to normal levels after 15 min and then progresses into a phase of inactivity (Yosten and Samson, 2009). Our findings are in agreement with these studies. Additionally, it has been found that nesfatin-1 is co-expressed with CRF in brain (Brailoiu et al., 2007; Fort et al., 2008). CRF expression alters responses to stress and regulates energy metabolism (Koob and Heinrichs, 1999; Kohno et al., 2008). Also, it has been reported that nesfatin-1 expression is mediated by alpha-MSH, and alpha-MSH administration provokes behaviors related to anxiety and fear (Merali et al., 2008). Therefore, it is possible that peripheral nesfatin-1 infusion exerted locomotor and metabolic effects via CRF and a-MSH signaling pathways in the brain.

Some results, especially those pertaining to food intake and feeding bouts in our long-term (7 day) study are very similar to the findings from the short-term (1 day) study. It was observed that nesfatin-1 suppresses food intake, average size of feeding bouts similar to findings of Goebel et al. (2011) and Stengel et al. (2012), and increases total activity even after 7 days of chronic infusion of nesfatin-1. In addition, the previous differences observed in RQ in the short-term study were transient, since they were not observed during the end of the long-term study. However, we did observe a consistent decrease in O_2_ consumption and CO_2_ production during the long-term study, which was specific to the light phase. This observation is also consistent with activation of energy sparing mechanisms and overall reduction of energy expenditure observed during the light phase in nesfatin-1 treated animals. In this scenario, it is possible that the sustained increase in the physical activity of nesfatin-1 treated rats may be masking potential differences in these variables during the dark phase.

Previously published results indicate that nesfatin-1 is reduced in circulation and in the regions of brain during food deprivation. We determined the NUCB2 mRNA in the whole brain, which encompasses a pool of abundant sources of nesfatin-1, and measured nesfatin-1 in serum collected from rat in various feeding conditions. It was found that food deprivation for 24 h causes a reduction in NUCB2 mRNA in the brain and stomach of rats, while re-feeding normalized NUCB2 mRNA expression in these tissues. Our data are in agreement with previous reports that 24-h of fasting downregulates NUCB2 mRNA expression in the stomach (Stengel et al., 2008; Gonzalez et al., 2011; Konczol et al., 2012; Wernecke et al., 2014) and in the PVN of rats (Oh-I et al., 2006). Fasting causes an urge to eat and during this, orexigenic an anorexigens signals are upregulated and downregulated, respectively. We observed that the reduction in NUCB2 mRNA expression was also followed by a corresponding decrease in circulating levels of nesfatin-1 in rats deprived of food for 24 h. These results confirm recent findings that plasma nesfatin-1 levels are significantly reduced after a 24 h fast (Stengel et al., 2009). In this study, Sprague Dawley rats were used and the circulating levels of nesfatin-1 was detected at 500 pg/mL. We found 3–5 ng/mL nesfatin-1 in Fischer 344 rats. These results are similar to what we have reported previously in fed Fischer 344 rats. It also agrees with the amount of nesfatin-1 detected by the nesfatin-1 ELISA manufacturer in undiluted plasma samples from rats. There are possible species and/or strain specific changes in circulating nesfatin-1. In addition, we also found elevated levels of NUCB2 in the circulation during the dark phase, 3 h (2200 h) in to the phase where the rats eat. These results strengthen the notion that nesfatin-1 is an endogenous, meal responsive anorexigen, and that the synthesis and release of this novel peptide are regulated by the metabolic status of the organism. It is possible that the antibody used in the ELISA detects both nesfatin-1 and its precursor forms. It is also unknown what percentage of the total measured NUCB2/nesfatin-1 immunoreactivity indeed represents nesfatin-1. Several other gut hormones, especially satiety peptides including CCK (Dockray, 2004), GLP-1 and PYY (Murphy et al., 2006) are released in a meal-sensitive fashion (Murphy and Bloom, 2006; Murphy et al., 2006). A postprandial increase in NUCB2 mRNA and circulating protein found in our studies further indicates that nesfatin-1 is released in response to nutrients. Whether nesfatin-1 is stimulated by diets and nutrients that constitute diets warrant future consideration. The abundant expression of NUCB2 in the gastrointestinal tract and its secretory profile similar to several other gut-derived satiety peptides reinforce the possibility that nesfatin-1 is an endogenous satiety signal.

Collectively, our results provide compelling evidence that nesfatin-1 is an anorexigen that influences various aspects of feeding and metabolism in rats. It highlights that the endogenous nesfatin-1 and its mRNA profiles befit an anorexigenic peptide in the strain of rats used here. The postprandial increase, and food deprivation based reduction in NUCB2 mRNA expression and circulating levels of NUCB2/nesfatin-1, all are suggestive of a meal responsive synthesis and secretion of this anorexigen. We found that peripheral administration of nesfatin-1 reduces feeding and increases physical activity in the dark phase without affecting body weight and fat mass, even after prolonged infusion. These effects are possibly dependent on the peptide dose, route of administration and animal strain, and are likely linked to the milieu of endocrine regulators targeted by nesfatin-1 in the dark vs. light phase. Further studies are required to distinguish doses that have appetite inhibitory vs. fat influencing effects alone, and the mechanisms that mediate such effects. Future research should focus on unraveling central and peripheral target sites, as well as the intracellular mechanisms by which nesfatin-1 elicits its metabolic actions. In addition, the peptidergic pathways that mediate the anorectic effects of nesfatin-1 also need to be studied.

## Author contributions

Conception and design of the experiments were done by SM and SU. SM, RG, RC, and SU were involved in the collection, analysis and interpretation of data. SM, RG, RC, and SU drafted the article or revised it critically for important intellectual content.

## Conflict of interest statement

The authors declare that the research was conducted in the absence of any commercial or financial relationships that could be construed as a potential conflict of interest.
