# Supplementary figures and images for: Long-term infusion of nesfatin-1 causes a sustained regulation of whole-body energy homeostasis of male Fischer 344 rats
Source: Front Cell Dev Biol. 2015 Apr 8;3:22. doi: 10.3389/fcell.2015.00022 (PMC4389570; doi:10.3389/fcell.2015.00022)

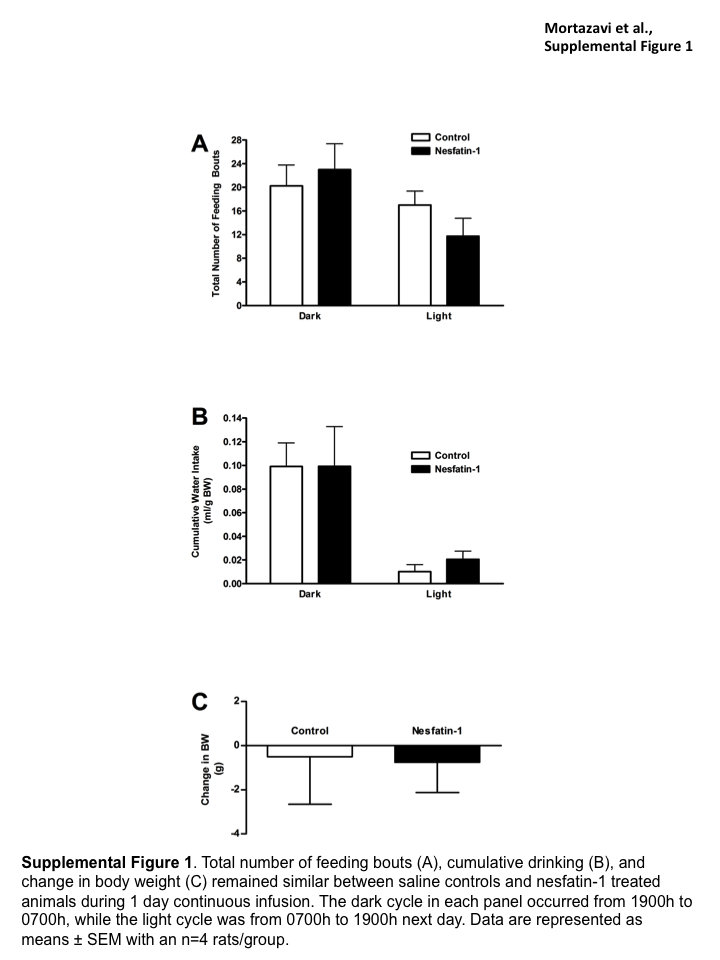

Supplement: Supplementary file 1 [file Image1.TIFF]

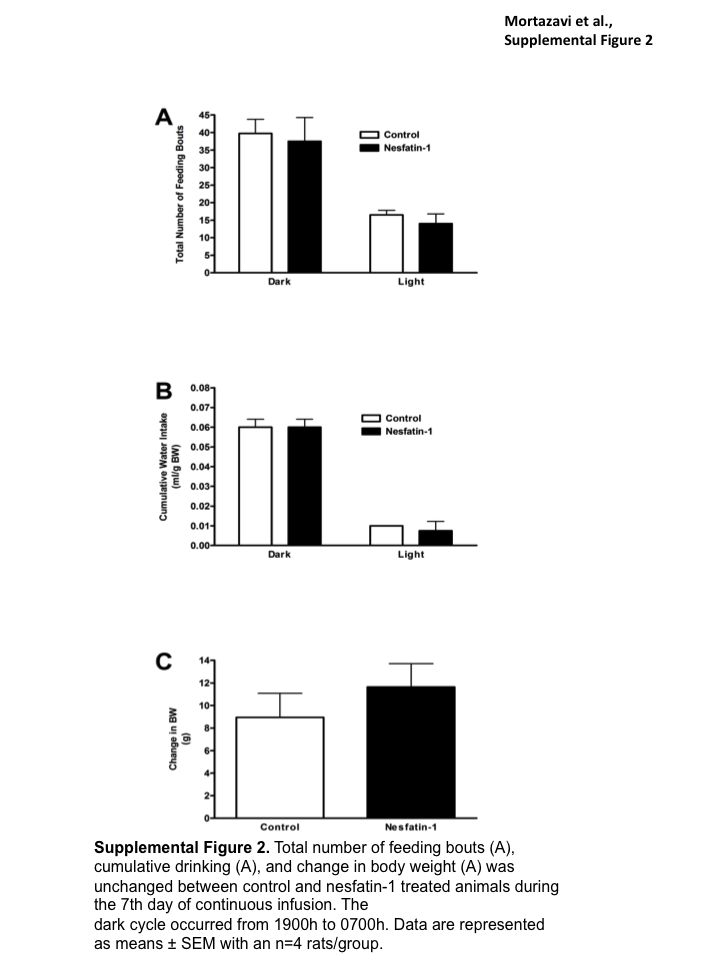

Supplement: Supplementary file 2 [file Image2.TIFF]
